# Supplementary material for: Identification of photoperiod-induced specific miRNAs in the adrenal glands of Sunite sheep (Ovis aries)
Source: Front Vet Sci. 2022 Jul 22;9:888207. doi: 10.3389/fvets.2022.888207 (PMC9354845; doi:10.3389/fvets.2022.888207)
Supplement: Supplementary file 1 [file Data_Sheet_1.ZIP › Supplementary file/miRDeep2 file.zip!/miRDeep2 file/Novel_Structure/SPLP42A3/Novel_186.pdf]

Provisional ID	: NC_019467.2_19108
Score total	: 2.1
Score for star read(s)	: -1.3
Score for read counts	: 0
Score for mfe	: 1.8
Score for randfold	: 1.6
Score for cons. seed	:
Total read count	: 115
Mature read count	: 115
Loop read count	: 0
Star read count	: 0

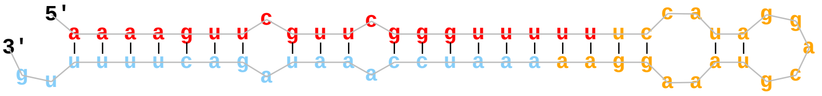

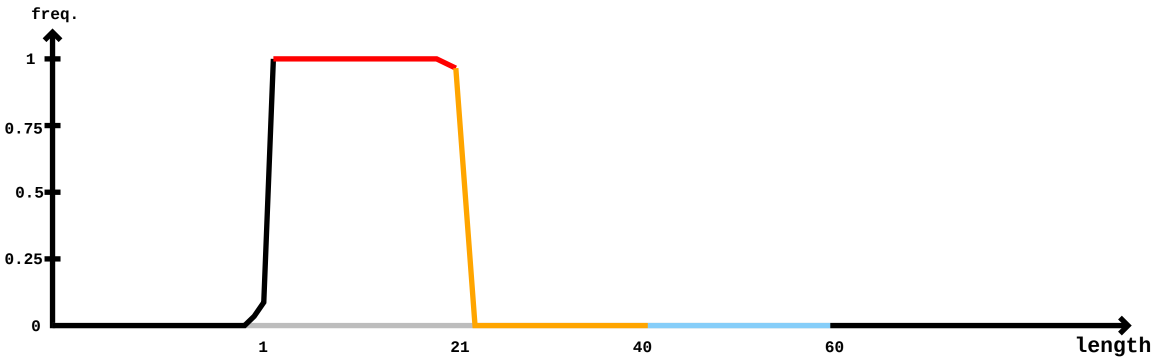

Mature **Star**

```
5'- uugaaaguauuggguuggcccaaaaguucguucggguuuuuuccauaggacguaaaggaaaaauccaaauagacuuuuuggccaacccaaucaauauauguugauguccc   -3'    exp
    ..........((((((((((.(((((((.(((.((((((((((..((.....))..)))))))))).))).))))))).))))))))))((((((....)))))).....    reads       mm          sample
    ...................cAaaaaguucguucggguuuu......................................................................    1           1           seq
    ...................cAaaaaguucguucggguuuuu.....................................................................    3           1           seq
    ....................caaaaguucguucggguuuu......................................................................    1           0           seq
    ....................Aaaaaguucguucggguuuuu.....................................................................    5           1           seq
    .....................aaaaguucguucggguuu.......................................................................    2           0           seq
    .....................aaaaguucguucggguuuuu.....................................................................    103         0           seq
```
